# Brainstorm: A User-Friendly Application for MEG/EEG Analysis

**DOI:** 10.1155/2011/879716

**Published:** 2011-04-13

**Authors:** François Tadel, Sylvain Baillet, John C. Mosher, Dimitrios Pantazis, Richard M. Leahy

**Affiliations:** ^1^Signal & Image Processing Institute, University of Southern California, Los Angeles, CA 90089, USA; ^2^MEG Program, Departments of Neurology & Biophysics, Froedtert & Medical College of Wisconsin, Milwaukee, WI 53226, USA; ^3^Epilepsy Center, Cleveland Clinic Neurological Institute, Cleveland, OH 44195, USA; ^4^MEG Lab, McGovern Institute for Brain Research, Massachusetts Institute of Technology, Cambridge, MA 02139, USA

## Abstract

Brainstorm is a collaborative open-source application dedicated to magnetoencephalography (MEG) and electroencephalography (EEG) data visualization and processing, with an emphasis on cortical source estimation techniques and their integration with anatomical magnetic resonance imaging (MRI) data. The primary objective of the software is to connect MEG/EEG neuroscience investigators with both the best-established and cutting-edge methods through a simple and intuitive graphical user interface (GUI).

## 1. Introduction

Although MEG and EEG instrumentation is becoming more common in neuroscience research centers and hospitals, research software availability and standardization remain limited compared to the other functional brain imaging modalities. MEG/EEG source imaging poses a series of specific technical challenges that have, until recently, impeded academic software developments and their acceptance by users (e.g., the multidimensional nature of the data, the multitude of approaches to modeling head tissues and geometry, and the ambiguity of source modeling). Ideally, MEG/EEG imaging is multimodal: MEG and EEG recordings need to be registered to a source space that may be obtained from structural MRI data, which adds to the complexity of the analysis. Further, there is no widely accepted standard MEG/EEG data format, which has limited the distribution and sharing of data and created a major technical hurdle to academic software developers.

MEG/EEG data analysis and source imaging feature a multitude of possible approaches, which draw on a wide range of signal processing techniques. Forward head modeling for example, which maps elemental neuronal current sources to scalp potentials and external magnetic fields, is dependent on the shape and conductivity of head tissues and can be performed using a number of methods, ranging from simple spherical head models [1] to overlapping spheres [2] and boundary or finite element methods [3]. Inverse source modeling, which resolves the cortical sources that gave rise to MEG/EEG recordings, has been approached through a multitude of methods, ranging from dipole fitting [4] to distributed source imaging using Bayesian inference [5–7]. This diversity of models and methods reflects the ill-posed nature of electrophysiological imaging which requires restrictive models or regularization procedures to ensure a stable inverse solution. 

The user's needs for analysis and visualization of MEG and EEG data vary greatly depending on their application. In a clinical environment, raw recordings are often used to identify and characterize abnormal brain activity, such as seizure events in epileptic patients [8]. Alternatively, ordering data into trials and averaging of an evoked response [9] remains the typical approach to revealing event-related cortical activity. Time-frequency decompositions [10] provide insight into induced responses and extend the analysis of MEG/EEG time series at the sensor and source levels to the spatial, temporal, and spectral dimensions. Many of these techniques give rise to computational and storage related challenges. More recently, an increasing number of methods have been proposed to address the detection of functional and effective connectivity among brain regions: coherence [11], phase locking value [12], Granger causality [13, 14] and its multivariate extensions [15], and canonical correlation [16] among others. Finally, the low spatial resolution and nonisotropic covariance structure of measurements requires adequate approaches to their statistical analysis [17]. 

Despite such daunting diversity and complexity in user needs and methodological approaches, an integrated software solution would be beneficial to the imaging community and provide progressive automation, standardization and reproducibility of some of the most common analysis pathways. The Brainstorm project was initiated more than 10 years ago in collaboration between the University of Southern California in Los Angeles, the Salpêtrière Hospital in Paris, and the Los Alamos National Laboratory in New Mexico. The project has been supported by the National Institutes of Health (NIH) in the USA and the Centre National de la Recherche Scientifique (CNRS) in France. Its objective is to make a broad range of electromagnetic source imaging and visualization techniques accessible to nontechnical users, with an emphasis on the interaction of users with their data at multiple stages of the analysis. The first version of the software was released in 2000, [18] and a full graphic user interface (GUI) was added to Brainstorm 2 in 2004 [19]. As the number of users grew, the interface was completely redesigned and improved, as described in this paper. In response to the high demand from users, many other tools were integrated in Brainstorm to cover the whole processing and visualization pipeline of MEG/EEG recordings, from the importing of data files, from a large selection of formats, to the statistical analysis of source imaging maps. Brainstorm 3 was made available for download in June 2009 and was featured at the 15th Human Brain Mapping Conference in San Francisco. The software is now being improved and updated on a regular basis. There have been about 950 new registered users since June 2009, for a total of 4,000 since the beginning of the project. 

Brainstorm is free and open source. Some recent publications using Brainstorm as a main analysis software tool are listed in [20–26]. This paper describes the Brainstorm project and the main features of the software, its connection to other projects, and some future developments that are planned for the next two years. This paper describes the software only; methodological background material is not presented here but can be found in multiple review articles and books, for example, [1, 27, 28].

## 2. Software Overview

Brainstorm is open-source software written almost entirely in Matlab scripts and distributed under the terms of the General Public License (GPL). Its interface is written in Java/Swing embedded in Matlab scripts, using Matlab's ability to work as a Java console. The use of Matlab and Java make Brainstorm a fully portable, cross-platform application. 

The advantage of scripting languages in a research environment is the simplicity to maintain, modify, exchange, and reuse functions and libraries. Although Python might be a better choice for a new project because of its noncommercial open source license, Brainstorm was built from a vast amount of pre-existing lines of Matlab code as its methodological foundations for data analysis. The Matlab development environment is also a high-performance prototyping tool. One important feature for users who do not own a Matlab license is that a stand-alone version of Brainstorm, generated with the Matlab Compiler, is also available for download for the Windows and Linux operating systems.

All software functions are accessible through the GUI, without any direct interaction with the Matlab environment; hence, Brainstorm can be used without Matlab or programming experience. For more advanced users, it is also possible to run all processes and displays from Matlab scripts, and all data structures manipulated by Brainstorm can be easily accessed from the Matlab command window.

The source code is accessible for developers on an SVN server, and all related Brainstorm files are compressed daily into a zip file that is publicly available from the website, to facilitate download and updates for the end user. Brainstorm also features an automatic update system that checks at each startup if the software should be updated and whether downloading a new version is necessary.

User documentation is mainly organized in detailed online tutorials illustrated with numerous screen captures that guide the user step by step through all software features. The entire website is based on a MoinMoin wiki system [29]; hence, the community of users is able to edit the online documentation. Users can report bugs or ask questions through a VBulletin forum [30], also accessible from the main website.

## 3. Integrated Interface

**Figure 1:**
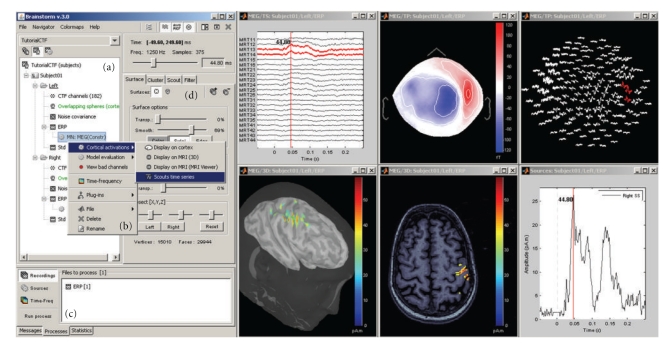
General overview of the Brainstorm interface. Considerable effort was made to make the design intuitive and easy to use. The interface includes: (a) a file database that provides direct access to all data (recordings, surfaces, etc.), (b) contextual menus that are available throughout the interface with a right-button click, (c) a batch tool that launches processes (filtering, averaging, statistical tests, etc.) for all files that were drag-and-dropped from the database; (right) multiple displays of information from the database, organized as individual figures and automatically positioned on the screen, and (d) properties of the currently active display.

Brainstorm is driven by its interface: it is not a library of functions on top of which a GUI has been added to simplify access but rather a generic environment structured around one unique interface in which specific functions were implemented (Figure 1). From the user perspective, its organization is contextual rather than linear: the multiple features from the software are not listed in long menus; they are accessible only when needed and are typically suggested within contextual popup menus or specific interface windows. This structure provides faster and easier access to requested functions.

Data files are saved in the Matlab.mat format and are organized in a structured database with three levels of classification: protocols, subjects, and experimental conditions. User data is always directly accessible from the database explorer, regardless of the actual file organization on the hard drive. This ensures immediate access to all protocol information and allows simultaneous display and comparison of recordings or sources from multiple runs, conditions, or subjects. 

## 4. Supported File Formats

Brainstorm requires three categories of inputs to proceed to MEG/EEG source analysis: the anatomy of the subject, the MEG/EEG recordings, and the 3D locations of the sensors. The anatomy input is usually a T1-weighted MRI of the full head, plus at least two tessellated surfaces representing the cerebral cortex and scalp. Supported MRI formats include Analyze, NIfTI, CTF, Neuromag, BrainVISA, and MGH. Brainstorm does not extract cortical and head surfaces from the MRI, but imports surfaces from external programs. Three popular and freely available surface formats are supported: BrainSuite [31], BrainVISA [32], and FreeSurfer [33].

The native file formats from three main MEG manufacturers are supported: Elekta-Neuromag, CTF, and BTi/4D-Neuroimaging. The generic file format developed at La Salpêtrière Hospital in Paris (LENA) is also supported. Supported EEG formats include: Neuroscan (cnt, eeg, avg), EGI (raw), BrainVision BrainAmp, EEGLab, and Cartool. Users can also import their data using generic ASCII text files.

Sensor locations are always included in MEG files; however, this is not the case for the majority of EEG file formats. Electrode locations need to be imported separately. Supported electrode definition files include: BESA, Polhemus Isotrak, Curry, EETrak, EGI, EMSE, Neuroscan, EEGLab, Cartool, and generic ASCII text files.

Other formats not yet supported by Brainstorm will be available shortly. Our strategy will merge Brainstorm's functions for the input and output from and to external file formats with the *fileio* module from the FieldTrip toolbox [34]. This independent library, also written in Matlab code, contains routines to read and write most of the file formats used in the MEG/EEG community and is already supported by the developers of multiple open-source software packages (EEGLab, SPM, and FieldTrip).

## 5. Data Preprocessing

Brainstorm features an extensive preprocessing pipeline for MEG/EEG data: visual or automatic detection of bad trials and bad channels, event marking and definition, baseline correction, frequency filtering, data resampling, averaging, and the estimation of noise statistics. Other preprocessing operations can be performed easily with other programs (EEGLab [35], FieldTrip, or MNE [36]) and results then imported into Brainstorm as described above.

Expanding preprocessing operations with the most popular techniques for noise reduction and automatic artifact detection is one of our priorities for the next few years of development.

## 6. Visualization of Sensor Data

**Figure 2:**
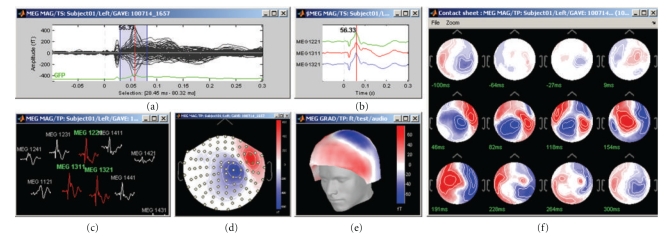
Brainstorm features multiple solutions for the visualization of MEG/EEG recordings.

Brainstorm provides a rich interface for displaying and interacting with MEG/EEG recordings (Figure 2) including various displays of time series (a)–(c), topographical mapping on 2D or 3D surfaces (d)-(e), generation of animations and series of snapshots of identical viewpoints at sequential time points (f), the selection of channels and time segments, and the manipulation of clusters of sensors. 

**Figure 3:**
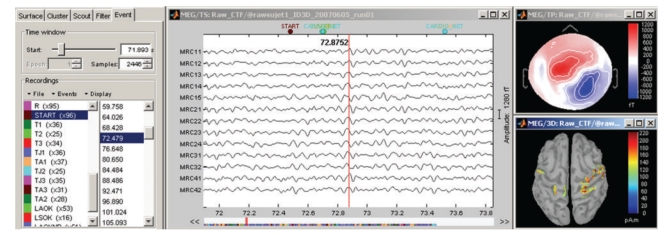
Interface for reviewing raw recordings and marking events.

These visualization tools can be used either on segments of recordings that are fully copied into the Brainstorm database and saved in the Matlab.mat file format, or on typically larger, ongoing recordings, directly read from the original files and which remain stored in native file formats. The interface for reviewing raw recordings (Figure 3) also features event marking in a fast and intuitive way, and the simultaneous display of the corresponding source model (see below).

## 7. Visualization of Anatomical Surfaces and Volumes from MRI

**Figure 4:**
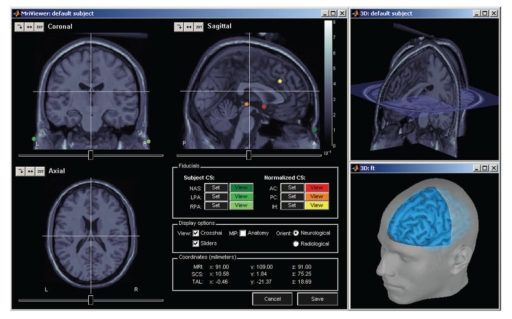
MRI and surface visualization.

Analysis can be performed on the individual subject anatomy (this requires the importation of the MRI and surfaces as described above) or using the Brainstorm's default anatomy (included in Brainstorm's distribution), which is derived from the MNI/Colin27 brain [37]. A number of options for surface visualization are available, including transparency, smoothing, and downsampling of the tessellated surface.  Figure 4 shows some of the possible options to visualize MRI volumes and surfaces.

## 8. Registration of MEG/EEG with MRI

Analysis in Brainstorm involves integration of data from multiple sources: MEG and/or EEG recordings, structural MRI scans, and cortical and scalp surface tessellations. Their geometrical registration in the same coordinate system is essential to the accuracy of source imaging. Brainstorm aligns all data in a subject coordinate system (SCS), whose definition is based on 3 fiducial markers: the nasion, left preauricular, and right preauricular points: more details regarding the definition of the SCS are available at Brainstorm's website.

**MRI-Surfaces**

**Figure 5:**
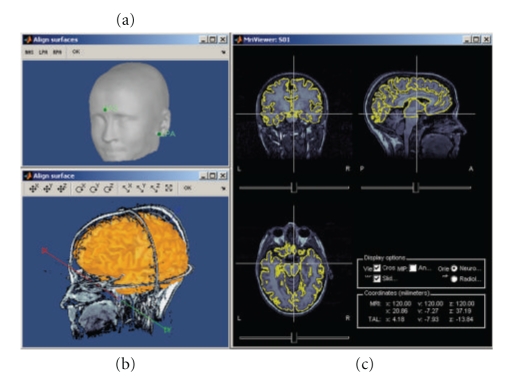
Registration of MRI data volumes with corresponding surface meshes.

Aligning the MRI data volume with the surface tessellations of the head tissues is straightforward and automatic as both usually originate from the same volume of data. Nevertheless, Brainstorm features several options to manually align the surface tessellations with the MRI and to perform quality control of this critical step including definition of the reference points on the scalp surface (Figure 5(a)) and visual verification of the proper alignment of one of the surfaces in the 3D MRI (Figures 5(b), 5(c)).

**Registration of MRI with MEG/EEG**

The fiducial reference points need to be first defined in the MRI volume (see above and Figure 4) and are then pair matched with the coordinates of the same reference points as measured in the coordinate system of the MEG/EEG during acquisition. Alignment based on three points only is relatively inaccurate and can be advantageously complemented by an automatic refinement procedure when the locations of additional scalp points were acquired during the MEG/EEG session, using a 3D digitizer device. Brainstorm lets the user run this additional alignment, which is based on an iterated closest point algorithm, automatically.

**Figure 6:**
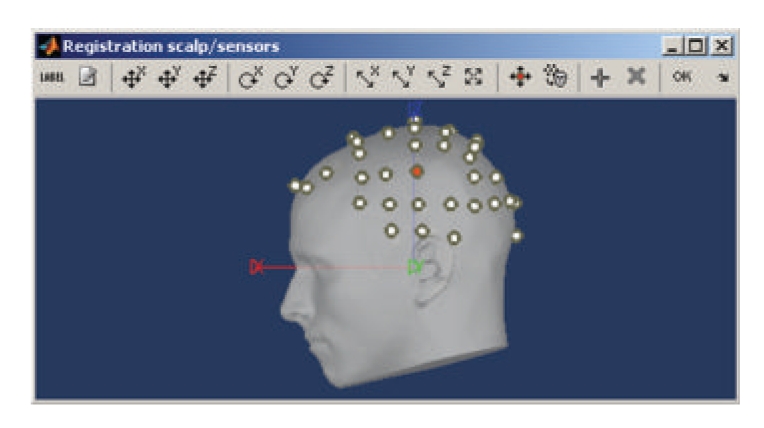
Brainstorm tool for editing of EEG electrode montages.

It is common in EEG to run a study without collecting individual anatomical data (MRI volume data or individual electrode positions). Brainstorm has a tool that lets users define and edit the locations of the EEG electrodes at the surface of the individual or generic head (Figure 6). This tool can be used to manually adjust one of the standard EEG montages available in the software, including those already defined for the MNI/Colin27 template anatomy.

**Volume and Surface Warping of the Template Anatomy**

**Figure 7:**
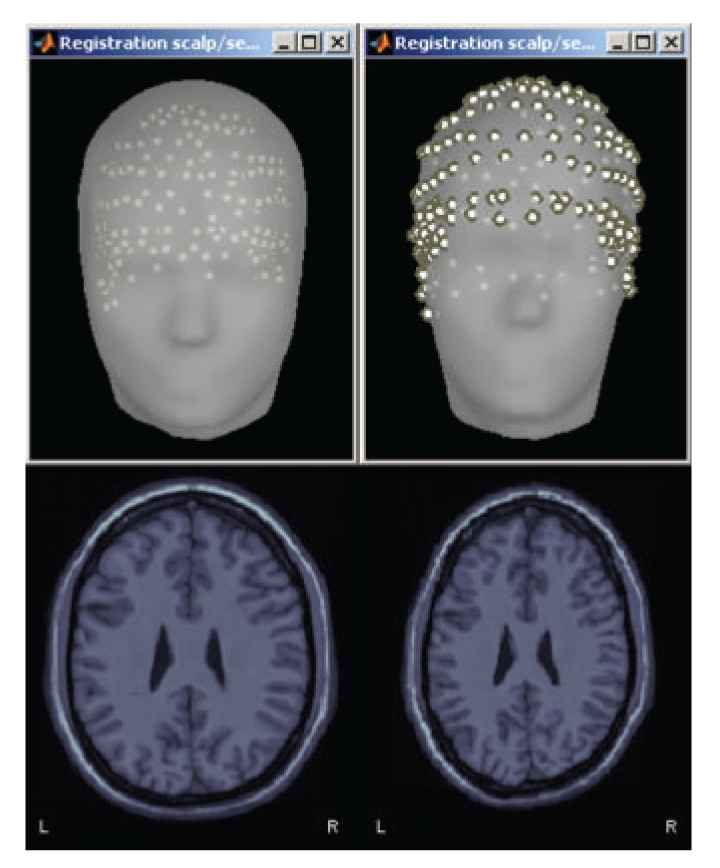
Warping of the MRI volume and corresponding tissue surface envelopes of the Colin27 template brain to fit a set a digitized head points (white dots in upper right corner): initial Colin27 anatomy (left) and warped to the scalp control points of another subject (right). Note how surfaces and MRI volumes are adjusted to the individual data.

When the individual MRI data is not available for a subject, the MNI/Colin27 template can be warped to fit a set of head points digitized from the individual anatomy of the subject. This creates an approximation of the individual anatomy based on scalp morphology, as illustrated in Figure 7. Technical details are provided in [38]. This is particularly useful for EEG studies where MRI scans were not acquired and the locations of scalp points are available.

## 9. Forward Modeling

Forward modeling refers to the correspondence between neural currents and MEG/EEG sensor measurements. This step depends on the shape and conductivity of the head and can be computed using a number of methods, ranging from simple spherical head models [1] to overlapping spheres [2] and boundary or finite element methods [39].

**Figure 8:**
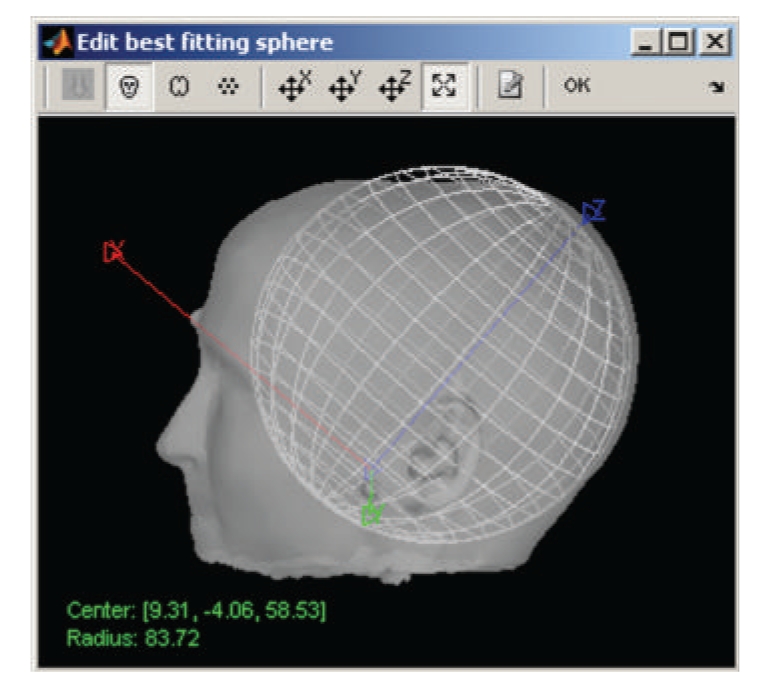
Interactive selection of the best-fitting sphere model parameter for MEG and EEG forward modeling.

Over the past ten years, multiple approaches to forward modeling have been prototyped, implemented, and tested in Brainstorm. The ones featured in the software today offer the best compromise between robustness (adaptability to any specific case) and accuracy (precision of the results). Other techniques will be added in the future. Current models include the single sphere and overlapping spheres methods for MEG [2] and Berg's three-layer sphere model for EEG [40]. For the single sphere methods, an interactive interface helps the user refine—after automatic estimation—the parameters of the sphere(s) that best fits the subject's head (Figure 8).

EEG is more sensitive to approximations in the geometry of the head as a volume conductor so that boundary element methods (BEMs) may improve model accuracy. A BEM approach for both MEG and EEG will soon be added to Brainstorm through a contribution from the OpenMEEG project [41], developed by the French National Institute for Research in Computer Science and Control (INRIA).

## 10. Inverse Modeling

Inverse modeling resolves the cortical sources that gave rise to a specific set of MEG or EEG recordings. In Brainstorm, the main method to estimate source activities is adapted from the depth-weighted minimum L2 norm estimator of cortical current density [42], which can subsequently be normalized using either the statistics of noise (dSPM [43]) or the data covariance (sLORETA [44]), as estimated from experimental recordings. For consistency and in an effort to promote standardization, the implementation of these estimators is similar to the ones available in the MNE software [36]. Two additional inverse models are available in Brainstorm: a linearly-constrained minimum variance (LCMV) beamformer [45] and the MUSIC signal classification technique [4, 46]. We also plan to add least squares multiple dipole fitting [4] to Brainstorm in the near future.

The region of support for these inverse methods can be either the entire head volume or restricted to the cortical surface, with or without constraints on source orientations. In the latter case, elementary dipole sources are distributed over the nodes of the surface mesh of the cortical surface. The orientation of the elementary dipoles can be left either unconstrained or constrained normally to the cortical surface. In all cases, the recommended number of dipoles to use for source estimation is about 15,000 (decimation of the original surface meshes can be performed within Brainstorm). 

Brainstorm can manage the various types of sensors (EEG, MEG gradiometers, and MEG magnetometers) that may be available within a given dataset. When multiple sensor types are processed together in a joint source model, the empirical noise covariance matrix is used to estimate the weight of each individual sensor in the global reconstruction. The noise covariance statistics are typically obtained from an empty-room recording, which captures the typical instrumental and environmental fluctuations.

## 11. Source Visualization and Analysis

**Figure 9:**
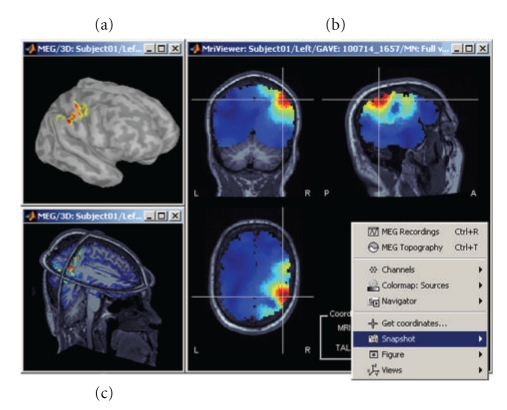
A variety of options for the visualization of estimated sources. (a) 3D rendering of the cortical surface, with control of surface smoothing; (c) 3D orthogonal planes of the MRI volumes; (b) conventional orthogonal views of the MRI volume with overlay of the MEG/EEG source density.

Brainstorm provides a large set of tools to display, visualize, and explore the spatio-temporal features of the estimated source maps (Figure 9), both on the cortical surface (a) and in the full head volume (b). The sources estimated on the cortical surface can be reprojected and displayed in the original volume of the MRI data (c) and on another mesh of the cortex at a higher or lower resolution. Reconstructed current values can be smoothed in space or in time before performing group analysis. 

**Figure 10:**
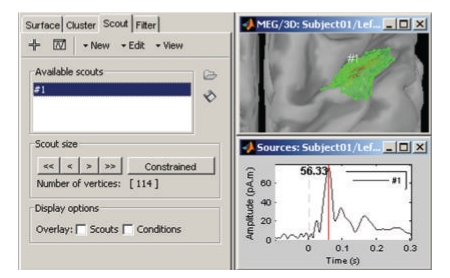
Selection of cortical regions of interest in Brainstorm and extraction of a representative time course of the elementary sources within.

A dedicated interface lets the user define and analyze the time courses of specific regions of interest, named *scouts* in Brainstorm (Figure 10). Brainstorm distribution includes two predefined segmentations of the default anatomy (MNI Colin27 [37]) into regions of interest, based on the anatomical atlases of Tzourio-Mazoyer et al. [47].

**Figure 11:**
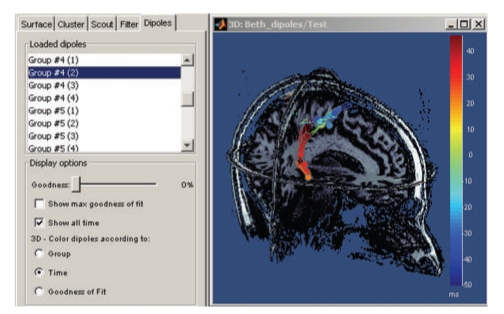
Temporal evolution of elementary dipole sources estimated with the external Xfit software. Data from a right-temporal epileptic spike. This component was implemented in collaboration with Elizabeth Bock, MEG Program, Medical College of Wisconsin.

The rich contextual popup menus available in all visualization windows suggest predefined selections of views for creating a large variety of plots. The resulting views can be saved as images, movies, or contact sheets (Figure 9). Note that it is also possible to import dipoles estimated with the FDA-approved software Xfit from Elekta-Neuromag (Figure 11). 

## 12. Time-Frequency Analysis of Sensor and Source Signals

Brainstorm features a dedicated user interface for performing the time-frequency decomposition of MEG/EEG sensor and source time series using Morlet wavelets [10]. The shape—scaled versions of complex-valued sinusoids weighted by a Gaussian kernel—of the Morlet wavelets can efficiently capture bursts of oscillatory brain activity. For this reason, they are one of the most popular tools for time-frequency decompositions of electrophysiological data [26, 48]. The temporal and spectral resolution of the decomposition can be adjusted by the user, depending on the experiment and the specific requirements of the data analysis to be performed.

Time-frequency decompositions tend to increase the volume of data dramatically, as it is decomposed in the space, time, and frequency dimensions. Brainstorm has been efficiently designed to either store the transformed data or compute it on the fly. 

Data can be analyzed as instantaneous measurements, or grouped into temporal and spectral bands of interest such as alpha (8–12 Hz) [26, 49], theta (5–7 Hz) [50–53], and so forth. Even though this reduces the resolution of the decomposition, it may benefit the analysis in multiple ways: reduced data storage requirements, improved signal-to-noise ratio, and a better control over the issue of multiple comparisons by reducing the number of concurrent hypothesis being tested. 

**Figure 12:**
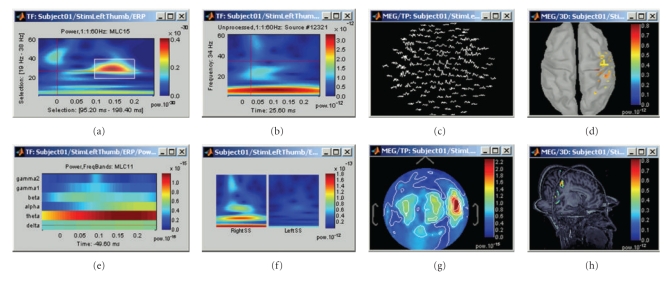
A variety of display options to visualize time-frequency decompositions using Brainstorm (see text for details).

Figure 12 illustrates some of the displays available to explore time-frequency decompositions: time-frequency maps of the times series from one sensor (a)-(b), one source (c) and one or more scouts (d), time courses of the power of the sensors for one frequency band (e), 2D/3D mappings (f), and cortical maps (g)-(h) of the power for one time and one frequency band. 

## 13. Graphical Batching Interface

**Figure 13:**
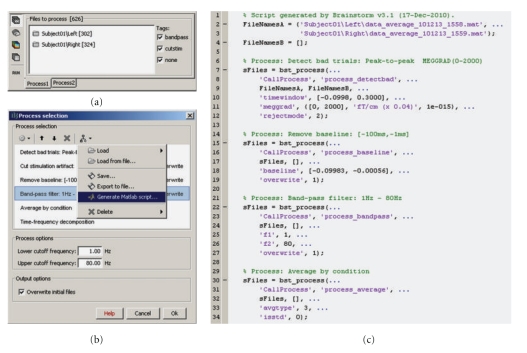
Graphical interface of the batching tool. (a) selection of the input files by drag-and-drop. (b) creation of an analysis pipeline. (c) example of Matlab script generated automatically.

The main window includes a graphical batching interface (Figure 13) that directly benefits from the database display: files are organized as a tree of subjects and conditions, and simple drag-and-drop operations readily select files for subsequent batch processing. Most of the Brainstorm features are available through this interface, including preprocessing of the recordings, averaging, estimation of the sources, time-frequency decompositions, and computing statistics. A full analysis pipeline can be created in a few minutes, saved in the user's preferences and reloaded in one click, executed directly or exported as a Matlab script.

The available processes are organized in a plug-in structure. Any Matlab script that is added to the plug-in folder and has the right format will be automatically detected and made available in the GUI. This mechanism makes the contribution from other developers to Brainstorm very easy.

## 14. High-Level Scripting

**Figure 14:**
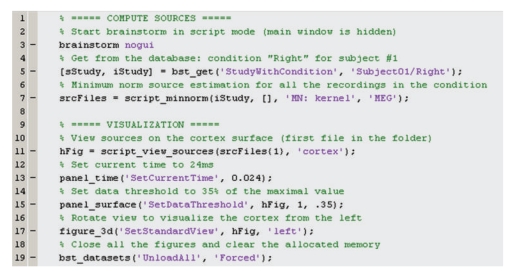
Example of Brainstorm script.

For advanced users and visualization purposes, Brainstorm can be used as a high-level scripting environment. All Brainstorm operations have been designed to interact with the graphical interface and the database; therefore, they have very simple inputs: mouse clicks and keyboard presses. As a result, the interface can be manipulated through Matlab scripts, and each mouse click can be translated into a line of script. Similar to working through the graphical interface, all contextual information is gathered from the interface and the database, so that most of the functions may be called with a limited number of parameters, and, for example, there is no need to keep track of file names. As a result, scripting with Brainstorm is intuitive and easy to use.  Figure 14 shows an example of a Matlab script using Brainstorm.

## 15. Solutions for Performing Group Analyses with MEG/EEG Data and Source Models

**Figure 15:**
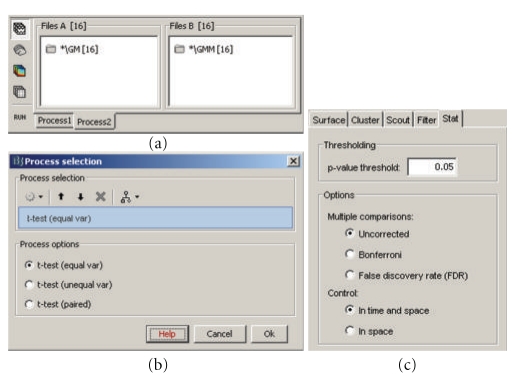
Student *t*-test between two conditions. (a) selection of the files. (b) selection of the test. (c) options tab for the visualization of statistical maps, including the selection of the thresholding method.

Brainstorm's “*Process2*” tab allows the comparison of two data samples. This corresponds to a single factor 2-level analysis and supported tests include simple difference, paired/unpaired Student *t*-tests of equal/unequal variance, and their nonparametric permutation alternatives [17]. The two groups can be assembled from any type of files, for example, two conditions within a subject, two conditions across subjects or two subjects for the same conditions, and so forth. These operations are generic in Brainstorm and can be applied to any type of data in the database: MEG/EEG recordings, source maps, and time-frequency decompositions. Furthermore, analysis of variance (ANOVA) tests are also supported up to 4 factors.  Figure 15 displays the use of a Student *t*-test to compare two conditions, “GM” and “GMM,” across 16 subjects.

We specifically address here how to perform multisubject data analysis using Brainstorm. In multisubject studies, measurement variance has two sources: the within-subject variance and the between-subject variance. Using collectively all trials from every subject simultaneously for comparisons is fixed-effects analysis [54] and does not consider the multiple sources of variance. Random-effects analysis [54, 55], which properly takes into account all sources of variance, is available in Brainstorm in its simplest and most commonly used form of the summary statistic approach [56, 57]. Based on this approach, analysis occurs at two levels. At the first level, trials from each subject are used to calculate statistics of interest separately for each subject, and at the second level, the different subjects are combined into an overall statistic. 

Consider the example of investigating experimental effects, where prestimulus data are compared against post-stimulus data. The first level analysis averages all trials from each subject to yield prestimulus and post-stimulus responses. The second-level analysis can be a paired *t*-test between the resulting *N* prestimulus maps versus the *N* post-stimulus maps, where *N* is the number of subjects. Brainstorm processes and statistics include averaging trials and paired *t*-tests, making such analysis possible. Also, the procedure described above assumes equal within-subject variance, but the subjects can be weighted accordingly if this is not the case.

Brainstorm also supports statistical thresholding of the resulting activation maps, which takes into account the multiple hypotheses testing problem. The available methods include Bonferroni, false discovery rate [58], which controls the expected portion of false positives among the rejected hypotheses, and familywise error rate [59], which controls the probability of at least one false positive under the null hypothesis of no experimental effect. The latter is controlled with a permutation test and the maximum statistic approach, as detailed in [17].

**Figure 16:**
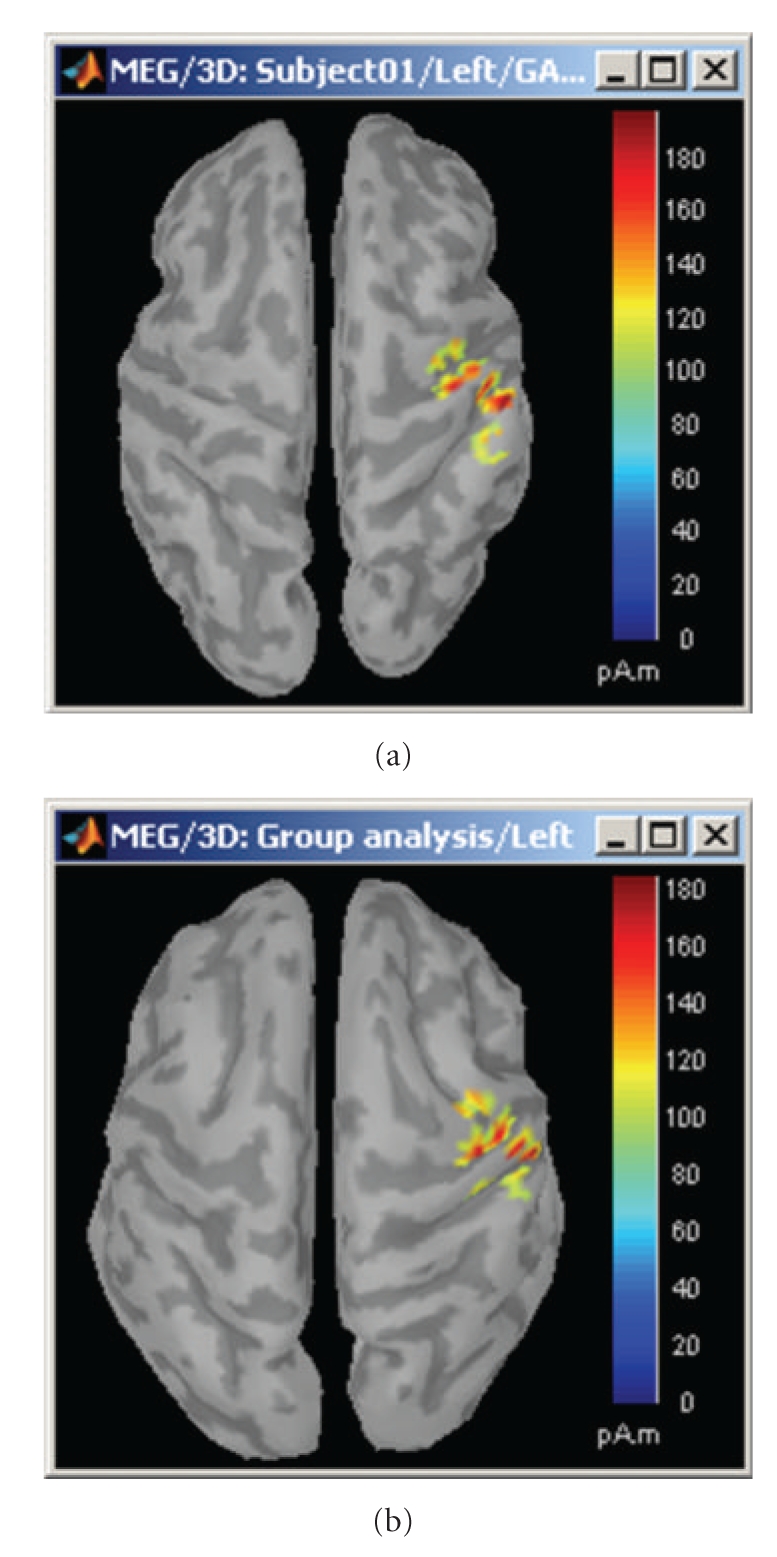
Cortical activations 46 ms after the electric stimulation of the left median nerve on the subject's brain (a) and their projection in the MNI brain (b).

In order to compare multiple subjects at the source level, an intermediate step is required if the sources were originally mapped on the individual subject anatomies. The sources estimated on individual brains are first projected on the cortical surface of the MNI-Colin27 brain. In the current implementation, the surface-to-surface registration is performed hemisphere by hemisphere using the following procedure: (1) alignment along the anterior commissure/posterior commissure axis, (2) spatial smoothing to preserve only the main features of the surfaces onto which the registration will be performed, (3) deformation of the individual surface to match the MNI surface with an iterative closest point algorithm (ICP) [60], and (4) interpolation of the source amplitudes using Shepard's method [61].  Figure 16 shows the sources on the individual anatomy (left), and its reprojection on the MNI brain (right). This simple approach will eventually be replaced by cortical surface registration and surface-constrained volume registration methods developed at the University of Southern California as described in [62]. We will also add functionality to use the common coordinate system used in FreeSurfer for intersubject surface registration.

## 16. Future Developments

Brainstorm is a project under constant development, and the current version provides an environment where new features are readily implemented and adapted to the interface. There are several recurrent requests from users for new features, as well as plans for future developments. Examples of forthcoming developments in the next two years include:

– expanding the preprocessing operations with the most popular techniques for noise reduction and automatic artifact detection,

– integration of methods for functional connectivity analysis and multivariate statistical analysis [16, 63],

– expanding forward and inverse calculations to include BEM and multiple dipole fitting methods,

– interface for simulating MEG/EEG recordings using simulated sources and realistic anatomy,

– segmentation of MEG/EEG recordings in functional micro-states, using optical flow models [64].

## 17. Brainstorm in the Software Development Landscape

Several commercial solutions to visualize and process MEG/EEG data are available. Most are developed for specific acquisition systems and are often designed by the manufacturers of these systems. They are typically unsuitable for research for several reasons: they are mainly driven by the requirements of clinical environment and FDA and CE certifications; their all-graphical interface seldom provides information about the underlying data analysis, file formats, are sometimes proprietary and undocumented; source code and description of the algorithms are not accessible to the user, and they are expensive. The research community needs solutions that are completely open, with the possibility of directly manipulating the code, data, and parameters.

As a result, many laboratories have developed their own tools for MEG and EEG data analysis. However, these tools are often not shared either because of the lack of interest or because of the required effort to support the software, develop documentation, and create and maintain a distribution website. However, the approach of developing individual tools is very limiting because of the limited availability of human resources assigned to software development in most research groups and the breadth of expertise that is required (electrophysiology, electromagnetic modeling, signal processing, statistics, classification, software optimization, real-time processing, human-machine interfaces ergonomics, etc.). 

In the past two decades, many projects have been developed to offer open and free alternatives to the wide range of commercial solutions. Common among these projects is the support by a large community of developers around the world, who produce free and reusable source code. For this purpose, the free software community equipped itself with tools to facilitate collaborative work, such as version managers, forums, wikis, and discussion lists. This approach to collaborative software development has not only reached a high level of maturity, but also proved its efficiency. The best example is probably the Linux operating system, whose stability matches or exceeds that of commercially produced operating systems. 

In the realm of functional brain mapping, open-source tools such as SPM [65] and EEGLab [35] have been broadly adopted in many research labs throughout the world. Providing open access to source code in combination with a willingness to accept additions and modifications from other sites clearly appeals both to users in clinical and neuroscientific research and others involved in methodology development. A variety of public licenses also allows developers to choose whether all or part of the code remains in the public domain. Importantly for software developed in academic and nonprofit labs, which are dependent on externally funded research support, recent experience indicates that open-source distribution is valued by the resesarch community and credit for this distribution is attributed to the original developers. 

Free software packages with similar features to Brainstorm (general purpose software for MEG/EEG) are EEGLab, FieldTrip, and MNE. The first two are written under the Matlab environment, with noncompiled scripts, and are supported by large communities of users connected with active forums and diffusion lists. EEGLab offers a simple but functional interface, and its target application is oriented towards the preprocessing of recordings and ICA analysis. FieldTrip is a rich and powerful toolbox that offers the widest range of functionalities, but without a graphic interface; its usage requires good skills in Matlab programming. MNE is also organized as a set of independent functions, easily scriptable and mostly oriented towards the preprocessing of the recordings and the source estimation using minimum norm technique, but written in C++ and compiled for Linux and MacOSX platforms. 

Brainstorm, in contrast, is an integrated application rather than a toolbox. At the present time, it offers fewer features than FieldTrip; but on the other hand, its intuitive interface, its powerful visualization tools, and the structure of its database allow the user to work at a higher level. It is possible to complete in a few minutes, and within a few mouse clicks, what would take hours otherwise: there is no need to write any scripts, and no need to think about where data files are stored on hard drives; the data is directly accessible, and a simple mouse click is sufficient to open a wide variety of display windows. It enables the researcher to concentrate on exploring his or her data. When visual exploration is complete and group analysis needs to be performed, Brainstorm offers a very high level scripting system, based on the interface and the database. The resulting code is easy to understand, and with few arguments: all the contextual information is gathered automatically from the database when needed, in contrast to FieldTrip, for example, where this information has to be specifically passed in arguments to each function.

To conclude, Brainstorm now represents a potentially highly-productive option for researchers using MEG or EEG; however, it is a work in progress and some key features are still missing. In the spirit of other open source developments, to the extent possible, we will reuse functions developed by other groups, which will then jointly maintain. Similarly, other developers are welcome to use code from Brainstorm in their software.
